# Genome wide comprehensive analysis and web resource development on cell wall degrading enzymes from phyto-parasitic nematodes

**DOI:** 10.1186/s12870-015-0576-4

**Published:** 2015-08-01

**Authors:** Krishan Mohan Rai, Vimal Kumar Balasubramanian, Cassie Marie Welker, Mingxiong Pang, Mei Mei Hii, Venugopal Mendu

**Affiliations:** Department of Plant & Soil Science, Texas Tech University, 2802, 15th street, Lubbock, TX 79409 USA; Current address Sarawak Biodiversity Centre, KM20, Jalan Borneo Heights, Semengoh, Locked Bag No. 3032, Kuching, Sarawak 93990 Malaysia

**Keywords:** Cell wall, Cell wall degrading enzymes, Cellulose, CWDEs, Database, Nematodes, Plant parasitic, Pectinases

## Abstract

**Background:**

The plant cell wall serves as a primary barrier against pathogen invasion. The success of a plant pathogen largely depends on its ability to overcome this barrier. During the infection process, plant parasitic nematodes secrete cell wall degrading enzymes (CWDEs) apart from piercing with their stylet, a sharp and hard mouthpart used for successful infection. CWDEs typically consist of cellulases, hemicellulases, and pectinases, which help the nematode to infect and establish the feeding structure or form a cyst. The study of nematode cell wall degrading enzymes not only enhance our understanding of the interaction between nematodes and their host, but also provides information on a novel source of enzymes for their potential use in biomass based biofuel/bioproduct industries. Although there is comprehensive information available on genome wide analysis of CWDEs for bacteria, fungi, termites and plants, but no comprehensive information available for plant pathogenic nematodes. Herein we have performed a genome wide analysis of CWDEs from the genome sequenced phyto pathogenic nematode species and developed a comprehensive publicly available database.

**Results:**

In the present study, we have performed a genome wide analysis for the presence of CWDEs from five plant parasitic nematode species with fully sequenced genomes covering three genera *viz. Bursaphelenchus, Glorodera* and *Meloidogyne.* Using the Hidden Markov Models (HMM) conserved domain profiles of the respective gene families, we have identified 530 genes encoding CWDEs that are distributed among 24 gene families of glycoside hydrolases (412) and polysaccharide lyases (118). Furthermore, expression profiles of these genes were analyzed across the life cycle of a potato cyst nematode. Most genes were found to have moderate to high expression from early to late infectious stages, while some clusters were invasion stage specific, indicating the role of these enzymes in the nematode’s infection and establishment process. Additionally, we have also developed a Nematode’s Plant Cell Wall Degrading Enzyme (NCWDE) database as a platform to provide a comprehensive outcome of the present study.

**Conclusions:**

Our study provides collective information about different families of CWDEs from five different sequenced plant pathogenic nematode species. The outcomes of this study will help in developing better strategies to curtail the nematode infection, as well as help in identification of novel cell wall degrading enzymes for biofuel/bioproduct industries.

**Electronic supplementary material:**

The online version of this article (doi:10.1186/s12870-015-0576-4) contains supplementary material, which is available to authorized users.

## Background

Plant parasitic nematodes employ physical and biochemical strategies for successful infection and establishment in host plants. Plant cells are surrounded by a cell wall, a rigid structure primarily made up of a dynamic network of matrix biopolymers along with different structural proteins [1–4]. The cell wall is an unique feature of plant cells which is not only important for maintaining their shape, size and growth, but also important for cell-to-cell and cell-to-environmental interactions [2]. The plant cell wall also acts as a primary defensive barrier against the attack of a plethora of plant pathogens *viz.* bacteria, viruses, fungi and nematodes [5–7]. Successful entry (infection) and survival (formation of syncytia or giant cell) of nematodes requires production of a battery of synergistically acting cell wall degrading enzymes. Among various plant pathogens, parasitic nematodes *Bursaphelenchus xylophilus* (pine wood nematode), *Globodera pallida* (potato cyst nematode), *Heterodera glycines* (soyabean cyst nematode) and different *Meloidogyne* species (root-knot nematodes) are responsible for the major crop damage and agricultural losses up to approximately $157 billion annually [8–10]. In order to establish the parasitic relation with the plants, most of these nematodes secrete a mix of synergistically active cell wall degrading enzymes (CWDEs) to invade the plant cell wall [11–13]. These enzyme mixes are administered into the plant cells after the physical damage by piercing them with a stylet, a hollow mouth spear like structure, present on the head of both ecto- and endo-parasites [14, 15].

The cell wall composition plays an important role in nematode-plant interactions [16]. Plant cell walls are mainly composed of cellulose (15–40 %), hemicellulose (30–40 %), lignin (20–30 %) and pectin biopolymers along with matrix proteins (1–5 %) [1–4]. Based on the cell wall composition, the nematode must produce a specific set of CWDEs, to degrade a host specific cell wall for successful entry into a plant species. Inability of a nematode to degrade any particular cell wall component may result in unsuccessful infection or survival in the host plant. It is plausible to alter the plant cell wall composition to make the cell walls recalcitrant to degradation by nematodes and thereby improve the plant’s resistance against nematodes. CWDEs have scientific and commercial importance, particularly in plant biomass based biofuel/bioproduct industries. The lignocellulosic material produced by plant biomass is utilized for the production of bioproducts and bioethanol via fermentation of cell wall derived sugars [17]. Lignocellulosic material often requires expensive physiochemical (steam & chemical) pretreatment to liberate sugar molecules for bioethanol production [18]. Efficient CWDEs are required for biological pretreatment to reduce the cost of physiochemical pretreatment and the associated chemical pollution. The CWDEs produced by bacteria and fungi have been characterized and used in the biofuel industry for biomass pretreatment [19]. However, the CWDEs produced by nematodes have not been explored for biofuel industrial applications. The enzymes produced by nematodes could provide a novel source of enzymes for the biofuel industry.

The very first experimental evidence of CWDEs presence in nematodes came with the identification of endogenous β-1,4-Endoglucanases (EC 3.2.1.4) in the esophageal glands of the cyst nematodes *G. rostochiensis* and *H. glycines* [20]. Subsequently, the endoglucanases were identified from different plant parasitic nematodes such as *B. xylophilus* (GH45) [21], *Ditylenchus africanus* and *Pratylenchus coffeae* (GH5) [22]. Different hemicelluloses and pectin degrading enzymes were also identified from plant parasitic nematodes using different bioinformatic and wet lab approaches [9, 20, 23, 24]. The first report of genome wide identification of CWDEs was from the very first sequenced plant parasitic nematode, *M. incognita* [9]. Furthermore, similar studies showed the presence of CWDEs from the genomes of *B. xylophilus* [25] and *G. pallida* [26]. Interestingly, the plant parasitic nematode *H. schachtii* produces a cellulose binding protein which interacts with the host’s pectin methyl esterase (PME) to modify the cell wall [27]. Over-expression of PME in transgenic *Arabidopsis thaliana* resulted in an increased nematode susceptibility, indicating that the nematode co-opts the host proteins for cell wall modification [27]. Hence, it is important to study CWDEs for developing effective strategies for plant defense apart from utilization in the biofuel industry.

The role of CWDEs in degrading the plant cell wall has been well studied from fungi, and various databases have been created harboring comprehensive information on plant cell wall degrading enzymes [28, 29]. A similar platform is not available for the nematodes, even though genome data is available for five major species of plant parasitic nematodes [9, 25, 26]. In most of the platforms providing such information, the nematode’s representation is limited to the model nematode, *Caenorhabditis elegans*. In the present study, we have collected the genomic resources from the completely sequenced plant parasitic nematode’s genomes and analyzed them for the presence of genes encoding CWDEs involved in degradation of the major cell wall components cellulose, hemicellulose and pectin. The identified CWDEs have been classified into a total of 24 gene families based on the HMM profile search using the CWDE families’ specific conserved sequences obtained from the Carbohydrate-Active enZymes (CAZy) database [30]. Various classes of cell wall related enzymes are defined by the CAZy database (http://www.cazy.org/). Carbohydrate Active enZymes (CAZymes) are involved in the biosynthesis/degradation/modification of glycoconjugates of oligo- and polysaccharides [29]. CAZymes are further classified in to Glycoside Hydrolases (GHs), Polysaccharide Lyases (PLs), Glycosyl Transferases (GTs), Carbohydrate Esterases (CEs) and enzymes with auxiliary activities (AAs) based on protein catalytic or functional domains [29, 30]. The CAZy database contains information about approximately 133 GH and 23 PL gene families. Cellulose and hemicellulose degrading enzymes belong to different families of the glycoside hydrolase class [11, 30]. The CAZymes produced by parasites play an important role in cell wall modification as well as host-pathogen interactions [30]. To understand the dynamic relation of these CAZymes, we further focused on expression profile of CWDEs during different stages of the life cycle of an endo-parasitic potato cyst nematode, *G. pallida*. We have found that CWDEs are expressed during the infection stage, and some CWDEs are induced during the infection and establishment stage, indicating their crucial role in nematode pathogenesis and survival in plants. The expression of CAZymes was dynamic and varied through the stages of the life cycle and the infection. Furthermore, we constructed a web-resource Nematode Cell Wall Degrading Enzyme database (NCWDE; http://www.pssc.ttu.edu/ncwde/index.html) as a platform to provide comprehensive information of all the plant CWDEs from the five species of genome sequenced plant parasitic nematodes. Apart from the identified CWDEs from this study, we have also included the CWDEs available in published literature from different species to expand the horizon of our database.

## Results and discussion

### Genome wide analysis of genes encoding plant cell wall degrading enzymes from five different nematode species

Genome sequencing of an organism provides comprehensive information on the presence of number of different genes, gene families and chromosomal locations. Here, for the analysis of CWDEs, we focused on the nematode species for which the whole genome sequence is available. Out of several species of plant pathogenic nematodes, the whole genome sequence is available for only five species from three genera (*B. xylophilus* [25], *G. pallida* [26], *M. floridensis*, *M. hapla* and *M. incognita* [9]) with a genome size ranging from 53.01 Mb (*M. hapla*) to 123.63 Mb (*G. pallida*). To perform genome wide analysis of CWDEs, a total of 90,314 protein sequences were downloaded from these five species with an average proteome size of 18,063 proteins per genome ranging from 14,420 (*M. hapla*) to 21,038 (*M. floridensis*) proteins (Table 1). The downloaded protein sequences were screened for the presence of proteins encoding plant cell wall degrading enzymes.

**Table 1:** Details of the plant pathogenic nematode species used to identify the CWDEs and construct database together with their pathogen specificity and feeding habits

Genus	Species	Name	Feeding Strategy	Genome Size (Mb)	Proteome size	CWDE’s Identified
*Bursaphelenchus*	*B. xylophilus*	Pine wood nematode	Stem/Bulb Nematodes	73.09	18,074	119
*Globodera*	*G. pallida*	Potato cyst nematode	Sedentary Endo-parasites	123.63	16,417	100
*Meloidogyne*	*M. floridensis*	Peach root-knot nematode	Sedentary Endo-parasites	96.67	21,038	102
	*M. hapla*	Northern root-knot nematode	Sedentary Endo-parasites	53.01	14,420	78
	*M. incognita*	Southern root-knot nematode	Sedentary Endo-parasites	82.1	20,365	131

**Fig. 1:**
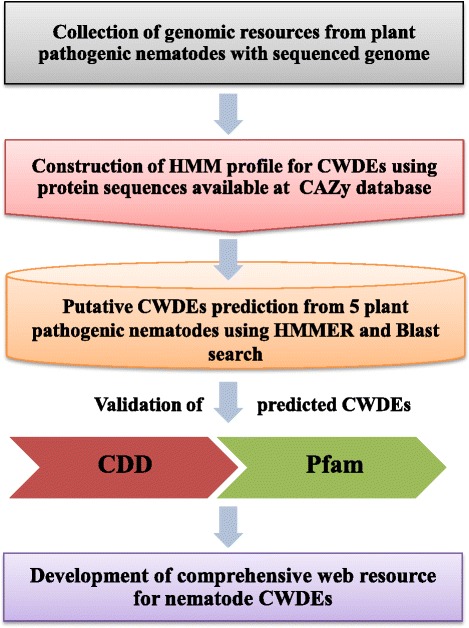
A schematic representation of the bioinformatic pipeline used to identify genes encoding CWDEs

Out of the total protein sequences analyzed against different databases, a total of 530 CWDE related protein sequences have been identified with an average of 106 CWDE related proteins from each species analyzed (Table 1, Additional file 1: Table S1). *M. hapla* was found to have a minimum number of CWDE encoding genes (78) whereas *M. incognita* was observed to have maximum number of CWDE encoding genes (131). Nevertheless, the number of CWDEs present per species showed no relation to the genome size. The present study showed a higher number of CWDE encoding genes than the previous individual reports on different nematode species [9, 25, 26]. Our analysis showed 119 CWDE encoding genes in *B. xylophilus* genome, in comparison to 73 genes identified in a previous study [25]. Similarly in *M. incognita* and *M. hapla*, we found 131 and 78 CWDE encoding genes in comparison to the reported 90 and 44 genes respectively [9]. The identification and distribution of the identified CWDE encoding proteins to the different gene families (Table 2) were performed using their signature domain profile constructed from the sequence information available on the CAZy database [11]. Additionally, the blast similarity search was also performed to identify CWDE encoding genes. After removing the redundant gene sequences, all the identified genes were further validated for the presence of related conserved domains using the Conserved Domain Database (CDD) and Protein families (Pfam). The bioinformatic pipeline used to identify CWDE genes has been illustrated in Fig. 1.

**Table 2:** Details of the identified CWDEs from plant pathogenic nematodes. Bx: *Bursaphelenchus xylophilus*, Gp: *Globodera pallida*, Mi: *Meloidogyne incognita*, Mh: *Meloidogyne hapla* and Mf: *Meloidogyne floridensis*

Substrate	CAZy Family	Activity	*Bx*	*Gp*	*Mi*	*Mh*	*Mf*
Substrate	CAZy Family	Activity	*Bx*	*Gp*	*Mi*	*Mh*	*Mf*
Ligno-Cellulose	GH3	β-Glucosidases	0	1	0	0	0
	GH5	Endo-β-1,4-glucanase/ cellulase	0	12	23	6	6
	GH7	Endo-β-1,4-glucanase	0	0	0	1	0
	GH45	Endoglucanase, endo-β-1,4-glucanase, cellulase	11	0	0	0	0
	GH27	α-Galactosidases	3	0	1	2	3
	GH31	α-Glucosidase	4	4	1	3	2
	GH35	β-Galactosidases	0	2	0	0	0
	GH38	α-Mannosidase (Class II)	7	7	1	2	3
	GH43	α-Arabinosidases	0	1	1	1	1
	GH47	Exo-acting α-1,2-mannosidases	5	4	6	4	2
	GH99	Endo-α-1,2-mannosidase	2	1	0	0	0
Chitin	GH75	β-1,4-chitosanases	0	0	1	1	2
	GH77	α-Amylase	0	0	1	0	0
	GH18	Chitinase	14	17	6	10	10
	GH19	Chitinase	2	0	0	0	0
	GH20	β-Hexosaminidase	8	3	5	3	2
Pectin	PL	Pectate lyase	15	8	36	22	37
1,3-Glucan	GH16	Xyloglucan:xyloglucosyltransferases	7	0	2	0	1
β-1,3-Glucans	GH64	β-1,3-glucanases	6	5	3	2	2
β-Glycans	GH2	β-Galactosidase	12	26	21	1	3
	GH15	Glucoamylase	2	0	0	0	0
	GH25	Lysozyme	17	1	20	16	23
	GH32	Invertase	0	8	1	2	3
	GH56	Hyaluronidase	4	0	2	2	2
	Total number of gene families		16	15	17	16	16
	Total number of CWDEs		119	100	131	78	102

**Fig. 2:**
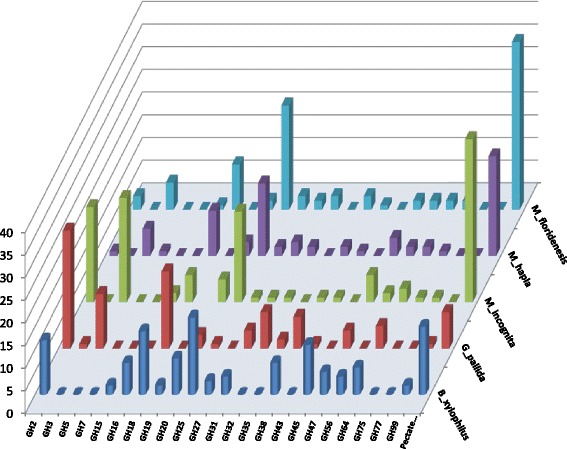
Species and family wise distribution of CWDE encoding genes identified from different species of plant pathogenic nematodes

**Fig. 3:**
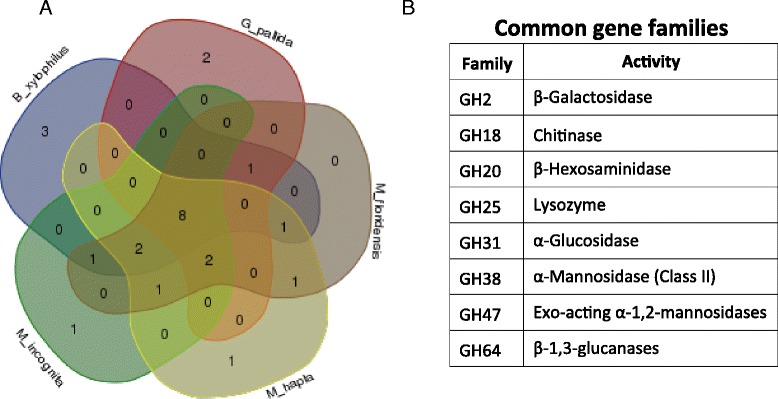
Comparative analyses of the gene families representing CWDEs from five different nematode species. **a** Venn diagram showing the number of common gene families identified between the different phyto-pathogenic nematode species. **b** List of common gene families identified in all the five species analyzed with their activities

### CAZy families, gene distribution and cell wall degrading enzymes

The identified CWDE genes have been classified into 24 gene families of CAZymes (Table 2 and Fig. 2), 23 related to glycoside hydrolases (GHs) whereas one is related to polysaccharide lyases (PLs). GHs are the enzymes responsible for hydrolyzing the glycosidic bond between carbohydrates or between a carbohydrate and non-carbohydrate moiety, whereas PLs are mainly responsible for the degradation of pectins and glycosaminoglycans [29]. All the analyzed nematode species showed a comparable total number of CAZy gene families, with a maximum of 17 in *M. incognita* and a minimum of 15 in *G. pallida* (Table 2). Though the total number of CAZy gene families is comparable among the five nematode species, the types of CAZy families identified are different (Table 2 and Fig. 2). Moreover, there is a variation in the number of CWDE genes present in each family between nematode species (eg. GH 27: 3, 0, 1, 2 & 3 in five different species). The distribution of genes per family varied within and between species indicating a possible evolution of plant parasitic nematodes according to their feeding behavior. The highest average number of CWDE genes per family was observed in *M. incognita* with an average of 7.70 genes per family, whereas the lowest of 4.88 genes per family was observed in *M. hapla* (Table 1). The identified 530 CWDEs from 24 families (glycoside hydrolases and polysaccharide lyases) were further classified, based on substrate specificity, into lignocellulases (cellulolytic, hemicellulolytic and lignolytic), pectinases, chitinases and other enzymes (Table 2 and Fig. 2).

#### Lignocellulases

The majority of plant biomass is composed of lignocellulosic material. Essentially, the lignocellulosic material is composed of cellulose, hemicellulose and lignin. Ferulic acid, a component of lignin, ester-links the cellulose and hemicellulose polysaccharides with lignin forming a complex lignocellulosic matrix of cell walls [31]. Cellulose is a polymer of β-(1,4)-linked glucose monomers and a major component of the plant cell wall [32]. Each cellulose synthase subunit synthesizes individual glucan chains with the glucose units arranged at 180 ° with respect to each other, hence, the repeating unit is cellobiose not glucose [32]. Glucose units of individual glucan chains form hydrogen bonds with the adjacent unit to produce a cellulose microfibril [33]. Cellulose fibrils are associated with other cell wall matrix polymers such as hemicellulose, pectin and lignin [34]. Cellulose is a homopolymer of glucose units while, hemicelluloses are heteropolymers with branched polysaccharides and hexose and pentose sugar monomers [35, 36]. Hemicelluloses are synthesized at the Golgi membranes and are exported to the cell wall for integration with other wall polysaccharides [37]. Hemicelluloses are composed of xyloglucan, xylans, mannans, glucomannans and mixed (β-1,4 & 1,3) glucans [38]. The composition of hemicelluloses differs between dicots and monocots and are classified as Type I and Type II cell walls respectively [2]. The primary cell walls of dicots contain a high proportion of Xyloglucans (XGs), while (glucurono)arabinoxylans (GAXs) are dominant in monocots [2, 35, 36, 38].

Cellulases are the enzymes responsible for the hydrolysis of native cellulose by breaking β-1,4 linkages in cellulose chains. The cellulose hydrolysis is achieved by the synergistic action of three types of cellulases: (1) endoglucanases (EC 3.2.1.4), (2) exoglucanases (EC 3.2.1.91), and (3) β-glucosidase (EC 3.2.1.21) [39]. In the present study, the CWDE encoding genes with this class of activity were mostly distributed into GH3 (β-Glucosidases), GH5 (Endo-β-1,4-glucanases), GH7 (Endo-β-1,4-glucanases) and GH45 (Endo-β-1,4-glucanases) families (Table 2 and Fig. 2). Among the cellulolytic gene families, GH5 and GH45 were found to have the most number of genes. Cellulose specific GH45 has been observed exclusively in the *B. xylophilus*, which is also in the agreement with the previous reports [21, 25] (Table 2). The family of GH45 cellulases showed high similarity to fungal genes and has not been found in any other nematodes indicating a possible horizontal gene transfer from fungi during the evolution of parasitism by nematodes [21, 25]. Hemicellulases are another important class of enzymes which degrade the second most abundant polymer of the cell wall i.e. hemicellulose. We also identified several gene families related to the hemicellulose specific activity *viz.* GH27 (α-Galactosidases), GH31 (α-Glucosidases), GH35 (α-Galactosidases), GH38 (α-mannosidase), GH43 (α-Arabinosidases), GH47 (Exo-acting α-1,2-mannosidases), and GH99 (Endo-acting α-1,2-mannosidases) (Table 2 and Fig. 2). GH31 with α-glucosides, GH38 with α-mannosidase (Class II) and GH47 with exo-acting α-1, 2-mannosidases activities were present in all of the five species analyzed (Fig. 3). Some of the GH3, GH5 and GH45 family enzymes were also reported to have the hemicellulase activity apart from the cellulose activity [40, 41]. There are reports of limited activity of GH5 family against the 1,4-β linked polysaccharides [41]. The GH45 family has been reported to have activity against the glucomannon in the pine wood nematode, *B. xylophilus* [40]. Similarly, GH16 with xyloglucan:xyloglucosyltransferases were also classified to have hemicellulose activity.

In addition to cellulose, hemicellulose and pectin, lignin is deposited in certain cell types, which synthesize secondary cell walls. Unlike cellulose and hemicelluloses which are made of sugars, lignin is a polymer of aromatic compounds. Lignin is a complex heteropolymer synthesized mainly from three aromatic alcohols *viz.* sinapyl, coniferyl and coumaryl alcohols [42, 43]. The monolignols are synthesized in the cytosol and are exported to the apoplast where the heteropolymer is synthesized from free radical coupling of monolignols [42, 43]. The proportion of different monolignols determines the lignin property and also varies from species to species depending on the tissue type, age, and environmental conditions [44]. In the present study, we also searched for the genes encoding enzymes, which can degrade lignin, an important component of the cell wall that makes the cell wall recalcitrant. Presence of lignin degrading enzymes makes the nematode degrade secondary cell walls that are rich in lignin content. According to the CAZy database classification, the lignin degrading enzymes belong to the multi-copper oxidase (AA1) family, which are classified as auxiliary activity (AA) enzymes [45]. Interestingly, we identified lignin degrading enzymes, laccase (Bux.s00116.660 and GPLIN_001134600) and laccase_like (GPLIN_001134500) from two nematodes i.e. pine wood and potato cyst nematodes (Additional file 2: Table S2). The presence of lignin degrading enzyme in root-knot nematodes indicates the specific need of lignin degrading enzymes in the pine wood nematode to invade the pine wood cell wall containing relatively high lignin content. Over all, the analysis showed the presence of a relatively large number of enzymes capable of degrading cellulose and hemicellulose compared to lignin. The nematodes primarily infect the root cells, which are mainly composed of primary cell walls rich in cellulose and hemicellulose. Absence of lignin degrading enzymes in majority of the nematode species could be due to the relatively less abundance or complete absence of lignin in the roots of the crop plants they infect. Enhancing the lignin content in the primary cell walls could be used as a strategy to enhance nematode resistance in crop plants.

#### Pectinases

Besides cellulose and hemicellulose, pectin constitutes the major component of the plant primary cell wall [35, 36]. Pectin is mainly located in the middle lamella and plays a major role in cell adhesion and wall porosity in association with cellulose and hemicelluloses [16, 35, 36]. Similar to hemicelluloses, pectic polysaccharides are synthesized in the Golgi apparatus as rhamnogalacturonan-I (RG-I), rhamnogalacturonan-II (RG-II) and homogalacturonan (HG) [35, 36]. A large fraction of total nematode CWDEs identified were found to be associated with Pectate Lyase (PL) activity, which is responsible for the degradation of another important component of cell wall i.e. pectin. Among the different nematodes species analyzed, the *Meliodogyne* species showed a higher number of pectin degrading (PL) enzymes (Table 2 and Fig. 2) 37 (*M. florigensis*), 22 (*M. hapla*) and 36 (*M. incognita*), whereas 8 and 15 were found in *G. pallida* and *B. xylophilus*, respectively. The presence of different types of pectin degrading enzymes (Table 3) in the secretion of plant pathogenic nematodes and their importance in maceration of plant roots during the nematode migration have been well reported [24, 46–48].

**Table 3:** List of published CWDE encoding genes from different species of plant pathogenic nematodes

Class	Gene/Protein	Nematode	References
Lignocellulose degrading enzymes	*BxEng1/2/3*	*B. xylophilus*	Kikuchi et al., 2004 [21]
	*DaEng1*	*Ditylenchus africanus*	Kyndt et al., 2008 [74]
	*HgEng1/2/3*	*H. glycines*	Smant et al., 1998 [20]; Yan et al., 1998 [75]; Yan et al., 2001 [76]; Gao et al., 2002a [77], 2002b [78]
	*HsENG1/2*	*H. schachtii*	De Meutter et al., 2001 [79]
	*GtEng1/2*	*G. tabacum*	Goellner et al., 2000 [65], 2001 [80]
	*GrEng1/2/3/4*	*G. rostochiensis*	Smant et al., 1998 [20]; Chen et al., 2005 [68]; Rehman 2009 [81]
	*MiEng1/2*	*M. incognita*	Rosso et al., 1999 [82]; Ledger et al., 2006 [83]
	*PcEng1*	*Pratylenchus coffeae*	Kyndt et al., 2008 [74]
	*PpEng1/2*	*P. penetrans*	Uehara et al., 2001 [84]
	*RsEng1A/1B/2/3*	*Radopholus similis*	Haegeman et al., 2008 [22]
	*RrEng1*	*Rotylenchulus reniform*	Wubben et al., 2010 [85]
	*BxEng1/2/3*	*B. xylophilus*	Shibuya and Kikuchi, 2008 [40]
	*MiXyl1/2/3*	*M. incognita*	Mitreva-Dautova, 2006 [86]; Haegeman et al., 2009 [87]
	*RsXyl1*	*R. similis*	Haegeman et al., 2009 [87]
Pectate Lyase	*BxPel1/2*	*B. xylophilus*	Kikuchi et al., 2006 [88]
	*GrPel1/2*	*G. rostochiensis*	Popeijus et al., 2000 [24]; Kundla et al., 2007 [47]
	*HgPel1*	*H. glycines*	De Boer et al., 2002 [89]
	*HsPel1/2*	*H. schachtii*	Vanholme et al., 2007 [48]
	*MiPel1/2*	*M. incognita*	Huang et al., 2005 [90]
	*MjPel1*	*M. javanica*	Doyle and Lambert 2002 [46]
Polygalactouronase	*MiPg1*	*M. incognita*	Jaubert et al., 2002 [91]

#### Chitinases

Chitin is a homopolymer of N-acetyl-β-D-glucosamine which is abundant in insect exoskeletons, fungal cell walls, nematode egg shells and some other biological matrices to provide support and increased strength to these structures [49]. Apart from the lignocellulosic and pectin degrading enzymes, we also found 85 genes distributed among five families known to have chitinase activity. Out of these five families *viz*. GH18 (Chitinases), GH19 (Chitinases), GH20 (β-Hexosaminidases), GH75 (β-1,4-chitosanases) and GH77 (α-amylases), GH18 and GH20 were the most abundant chitinases found across all the five genomes analyzed (Table 2 and Figs. 2 and 3). The existence of 42 genes comprising chitinase and N-acetylglucosaminidase activity has also been reported in the free-living nematode, *C. elegans* [50]. Nematode chitinases play an important role in remodeling the egg shell chitin during the nematode development [51]. Additionally, the presence of chitinase enzymes in nematodes may have a role in utilizing the fungus and insect derived chitin polymers present in the soil as an additional nutritional source. Chitinase enzymes could also help nematodes to feed on the soil fungi. It has been reported that the soil inhabiting nematode, *Filenchus* species, reproduce by feeding on fungi in soil [52]. It is considered that the plant-parasitic nematode species, *Tylenchida*, is evolved from ancestral fungal feeding nematodes [52] suggesting that these genes are evolutionarily conserved in this nematode species. These enzymes might be involved in their defense against the nematophagous fungi in soil.

#### Other enzymes

Apart from these enzymes, we also identified gene families with lysozyme (GH25) and invertase (GH32) activity. Invertase plays an important role in catalyzing the conversion of the abundant plant sugar, sucrose into the monosaccharides glucose and fructose, which then can be utilized as a carbon source by plant parasitic nematodes [9]. Overall, the wide host range and substrate specific CWDEs present in these plant parasitic nematodes have been reported to play important roles while establishing the host-pathogen interaction [9, 25, 53].

### Auxiliary Activity (AA) enzymes

Auxiliary activity (AA) enzymes are the redox enzymes that work synergistically with the other carbohydrate active enzymes. With the recent discoveries in this area, CAZy database added AA enzymes with 13 sub-classes as a new class of enzymes to expand its horizon [45]. Mining of all the five nematode genomes for the presence of AA class of enzymes showed presence of seven genes encoding multi-copper oxidase (AA1), seven genes encoding GMC oxido-reductase (AA3), single gene encoding vanillyl alcohol oxidase (AA4) and three genes encoding Gluco-oligosaccharide oxidase (AA7) were present in the plant parasitic nematodes (Additional file 2: Table S2). The sub-class AA1, a multi-copper oxidase has been reported to play important role in the lignin degradation. Interestingly, seven genes were identified from this sub-class out of which, two were classified as laccase (EC 1.10.3.2) (Bux.s00116.660 and GPLIN_001134600) whereas one as laccase_like enzyme (EC 1.10.3.2) (GPLIN_001134500) (Additional file 2: Table S2). AA1 sub-class has been reported to be present in the fungal genomes especially in Ascomycota and plays diverse roles including lignin degradation and plant-pathogenic interactions [54]. The analysis identified seven genes related to the sub-class AA3 [glucose-methanol-choline (GMC) oxido-reductase] from all the five plant parasitic nematodes indicating the importance of AA3 possible accessory role played by these enzymes (Additional file 2: Table S2). The AA3 enzymes are flavoproteins containing a flavin-adenine dinucleotide (FAD)-binding domain reported to play a role in cellulose, hemicellulose and lignin biodegradation [55, 56]. The sub-class AA3 has also been reported in lignocellulose-degrading fungi to produce an extracellular hemoflavoenzyme, cellobiose dehydrogenases (EC 1.1.99.18) under the cellulolytic culture conditions [56]. Apart from these sub-classes, one gene encoding vanillyl alcohol oxidase (AA4) and three genes encoding Gluco-oligosaccharide oxidase (AA7) were also identified in the present study from *B. xylophilus* and *G. pallida*, respectively. AA4 sub-class has been reported to be active on intermediate aromatic compounds produced during the lignin degradation [45]. Similarly sub-class AA7 has been reported to oxidize the different carbohydrates such as D-glucose, maltose, lactose, cellobiose, malto- and cello-oligosaccharides and also play role in detoxification/biotransformation of lignocellulosic materials [45, 57]. Apart from the sub-classes AA1 and AA3, AA6, AA8 and AA9 have been also reported in different phyto-parasitic fungal genomes [45] which indicates the significant role of AA class of enzymes in establishing the plant-pathogen interaction by aiding the cell wall degrading enzymes.

### Carbohydrate Binding Modules (CBMs)

The Carbohydrate Binding Modules (CBMs) are non-catalytic domains known to associate with the catalytic domains of the CWDEs and help in enhancing the activity of the catalytic domains [58, 59]. A total of 71 CBM families based on the sequence similarity have been listed in the CAZy database [30]. These CBMs are reported to display variation in the ligand specificity and have been shown to recognize various carbohydrate moieties such as crystalline cellulose, non-crystalline cellulose, chitin, β-1,3-glucans and β-1,3-1,4-mixed linkage glucans, xylan, mannan, galactan and starch [58]. Since these modules play an important role in the CWDEs, genome wide analysis was performed for the presence of CBM modules that resulted in the identification of four classes of CBMs (CBM2, CBM14, CBM20 and CBM21) in the nematode genomes. Of the four modules, only two, CBM2 and CBM14 were found to be associated with GH5 and GH18 families of CWDEs (Additional file 2: Table S3). A total of 18 genes were identified related to the CMB2 sub-class, 13 of which belong to the *M. incognita* whereas 4 and 1 genes belong to the *G. pallida* and *M. hapla,* respectively. Similarly, out of four genes from the CBM14 sub-class, each of the analyzed nematodes has a single gene except the *M. incognita* (Additional file 2: Table S3). The CBMs have been previously reported to be associated with the plant-pathogenic fungi [60] as well as plant-parasitic nematodes [61]. CBM protein has been shown to interact with a host pectin methylesterase (PME) in *H. glycine* [27]. Since the PME has been reported to involve in the regulation of cell growth and expansion, the *H. glycine* CBM was hypothesized to have role in the syncytium expansion [27].

### Expression profile of CWDE genes during the nematode’s life cycle

**Fig. 4:**
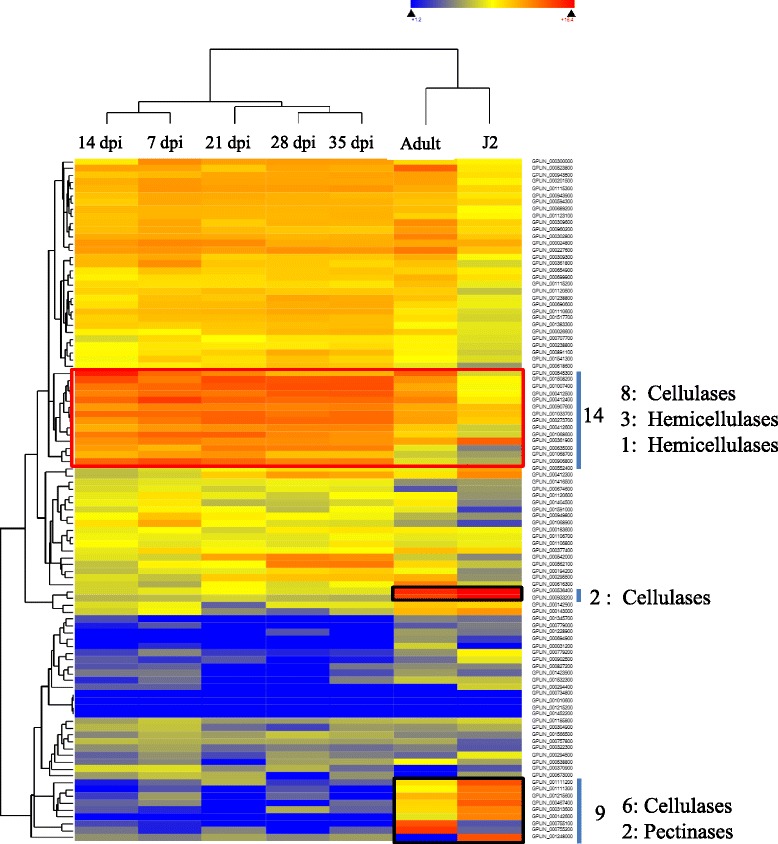
Heat map showing hierarchical clustering of CWDEs across different stages of the life-cycle of potato cyst nematode, *G. pallida.* The expression of genes has been shown in different colors. *Blue color* indicates the down-regulated genes; *yellow color* indicates the moderately expressed genes, whereas the *red color* indicates the highly expressing genes

Genome wide expression analysis will provide information on the genes that are expressed at a specific stage of development or in a particular condition while the whole genome sequence provides comprehensive information on the total number of genes present in an organism. The plant CWDEs produced by plant pathogenic nematodes have been shown to play an important role in establishing the parasitic relationship with plants during the infection process [9, 10, 25, 26, 62, 63]. Timely expression of these genes is essential to establish the infection process by degrading the cell walls for an easy entry and establishment. Endoglucanases *HgEng1* and *HgEng2,* and *GtEng1* and *GtEng2* are expressed during the penetration and intracellular migration of J2 within roots of the soybean cyst nematode and the tobacco cyst nematode, respectively [64, 65]. Apart from transcript level evidences, the soybean cyst nematode protein, HgENG2, has been shown to be synthesized from the sub-ventral esophageal gland cells of the nematode and secreted into the soybean root tissue using immunolocalization studies [66]. It has been shown that HgENG2 is being secreted from the stylet during their migratory path after the 24 h of inoculation [66]. To further interpret the role of CWDE genes in plant pathogenic nematodes, the expression analysis of these CWDE encoding genes in different stages of plant parasitic nematode life cycle was analyzed. The publically available transcriptome repository (SRA: Short Read Archive dataset) has been searched for the transcriptome data covering different stages of a nematode life cycle. The potato cyst nematode, *G. pallida* was the only nematode for which a comprehensive transcriptome data is available for the entire life cycle i.e. invasive larval stage J2, adult male, 1, 7, 14, 28 and 35 days post infection (dpi). The data was downloaded and analyzed the expression of 100 CWDE genes identified from the *G. pallida* using. (Additional file 2: Table S4). Most of the CWDE genes identified were expressed during different stages of the nematode’s lifecycle (Fig. 4, Additional file 2: Table S5). The expression profile of the CWDE genes could be clustered into nine major clusters using the hierarchical clustering analysis with the Euclidean distance method of the DNASTAR QSeq software. Most of the cluster 1 and 2 genes were among the moderately high expressing genes across all the stages of the life-cycle except for the J2 stage, where these genes have moderate expression. All the genes of cluster three were among the highly expressed genes across the early to later stages of infection. Out of the 14 genes of this cluster, eight are related to cellulose degradation and four were responsible for hemicellulose degradation (Fig. 4). The high level of expression of these genes in the early and later stage of infection is also supported by the endo-parasitic feeding habit of this cyst nematode [10]. The GHs and PLs are required for the degradation of cell wall components to invade, to migrate into the cell or to dissolve the cell wall for syncytium formation [67]. The importance of cell wall degrading enzymes for the nematode’s parasitic relationship has been shown by RNAi knock-down of genes with cellulose activity in the potato cyst nematode *G. rostochiensis* [68]. Silencing of β-1,4-endoglucanase reduced the ability of the cyst nematode to infect the potato roots [68], demonstrating the importance of the CWDEs in successful infection of crop plants. It is possible to develop resistant crops by altering the composition of cell walls to make them recalcitrant to degradation by nematode CWDEs. Among the other clusters, cluster 5 and 6 related genes have moderate expression throughout the stages, whereas most of the cluster 7, 8 and 9 related genes were repressed or had very little expression. Although almost all of the cluster nine genes were repressed, a group of nine genes (six cellulases, and two pectate lyase) were observed to have strikingly higher expression in J2 or the adult stage of the life-cycle (Fig. 4). The specific expression of these genes during the infective J2 stage suggests that they possibly have an important role in plant cell invasion during the infection by parasitic nematodes.

### Nematode’s Cell Wall Degrading Enzyme database (NCWDE)

**Fig. 5:**
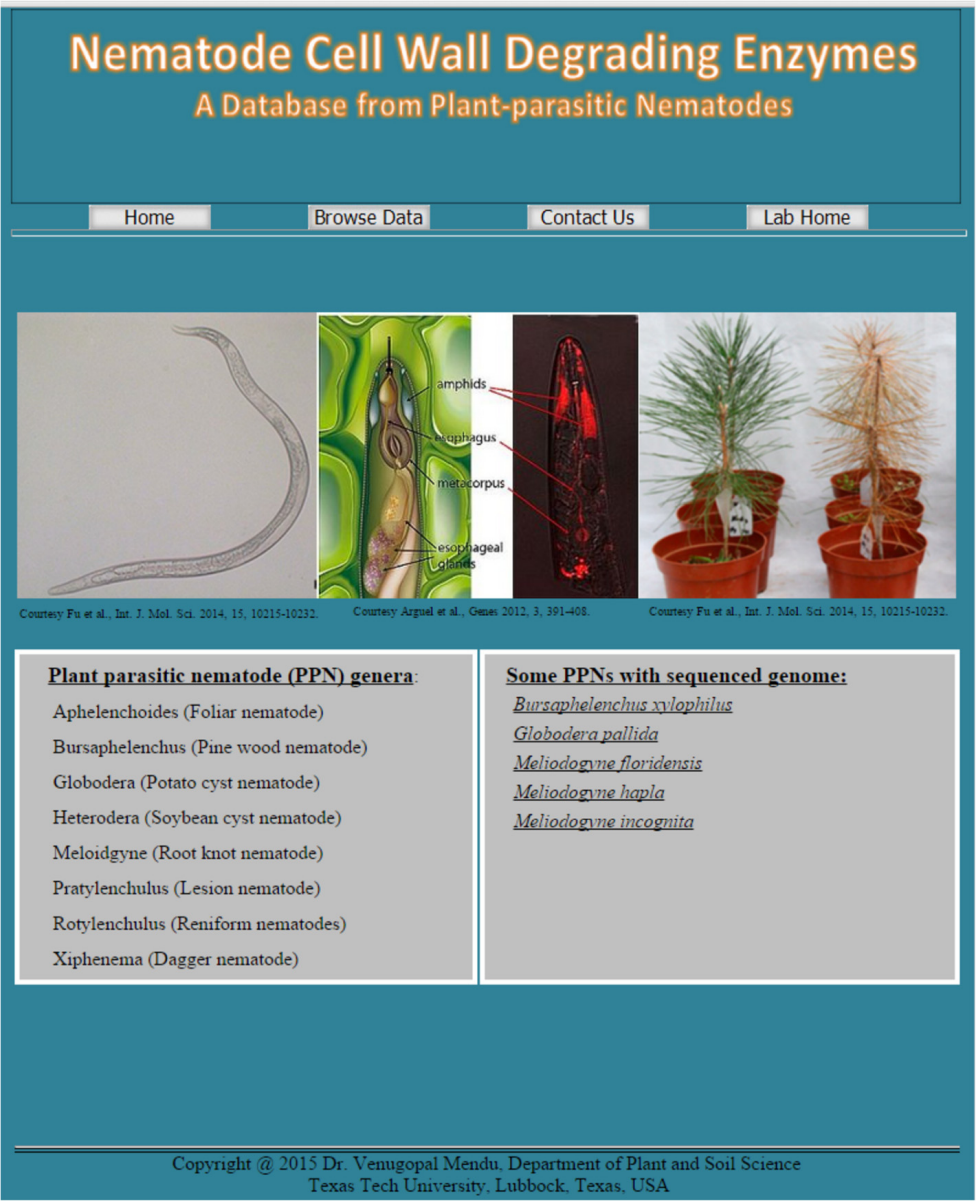
A screenshot representation of the nematode’s cell wall degrading enzyme database web page

All the available databases for CWDEs (CAZy and fungal plant cell wall degrading enzyme database) [11, 28] have a vast amount of information about these genes, but information about the nematode’s CWDEs is mostly limited to the *C. elegans*. To ensure collective and easy access of information related to nematode CWDEs, we constructed a Nematode’s Cell Wall Degrading Enzyme database (NCWDE): a web resource which provides comprehensive information related to the CWDEs from the plant parasitic nematodes (Fig. 5). Apart from the genome wide identification of the CWDEs from the five nematode genomes, we searched the available literature for the individual CWDEs from the different nematode species for which genome data are not available, and incorporated them into the database to ensure their representation (Table 3). All the information related to these databases is available for public access at http://www.pssc.ttu.edu/ncwde/index.html.

### Importance of nematodes in ecology, environment, biofuels and soil health

Though plant parasitic nematodes are responsible for significant agricultural losses by devastating a wide range of economically important crops, the presence of these potential pathogens in particular crop land can be utilized as the bio-indicator for monitoring and managing the soil ecosystem i.e. soil health, the ecological balance and environmental status [69]. The soil health, one of the important factors responsible for the crop productivity, is inter-related to the ecological and environmental status of that area *viz*. microbial diversity, pollutants and heavy metals [70]. Interestingly, the plant pathogenic nematodes can regulate the microbial diversity of any crop field by overgrazing a specific bacterial or fungal population or promoting them either by releasing growth limiting nutrients or disseminating specific microbial propagules to the soil [69, 71]. The resulting microfaunna of that cropland will affect their nutrient recycling ability, the factor directly associated with crop productivity. Eventually, analyzing the CWDE profile of plant parasitic nematodes will provide their feeding preferences and will help in assessing what kind of nutrients are required to maintain the soil health or long term effects of nematode presence in soil. Another important outcome of the nematode CWDE study is discovering novel enzymes for bioproduct/biofuel production. The biopolymers present in plant cell walls, such as cellulose and xylans, are rich sources of hydrocarbon for the production of biofuels, particularly bioethanol [12]. The recalcitrant behavior of the lignocellulosic biomass and the high cost of the required hydrolytic enzymes are major hurdles in the successful utilization of this abundantly available resource. The CWDEs identified in the present study have shown a wide range of substrate specificity (cellulose, hemicellulose, lignin, pectin, etc.). Considering the wide substrate coverage, these plant pathogenic nematode derived CWDEs may be utilized for more efficient degradation of the complex plant biomass. The nematode CWDEs present a novel source of enzymes because of their ability to function *in planta*, compared to the widely used fungal or bacterial enzymes.

## Conclusions

In the present study, we have performed a comprehensive analysis of CWDEs from plant parasitic nematodes with sequenced genomes and have developed a database to provide the comprehensive information related to CWDEs. Although primary focus of this study was to identify the genes encoding plant CWDEs, we also identified the CWDEs of bacterial and fungal origin due to horizontal gene transfer of these genes from bacteria and fungi to nematodes. Our results showed the presence of common CWDEs across all the species, as well as, the presence of some species-specific CWDEs. Moreover, the presence of differential, as well as, ubiquitous expression clusters of these genes in different stages of the cyst nematode *G. pallida,* suggest that these enzymes play an important role throughout the life-cycle of nematodes. The small number of genome sequenced species limits our present study, but with the availability of more sequenced plant pathogenic nematode genomes in the future, we will expand the database. The importance of CWDEs is not only limited to the establishment of a parasitic relationship with the host species, but they are also good sources for novel and potentially more efficient enzymes to degrade recalcitrant plant cell walls for their use in biofuel/bioproduct industries. Furthermore, the NCWDE database provides information for the functional characterization of these enzymes in nematodes by forward and reverse genetic methods which can eventually be used to develop nematode resistant crops.

## Methods

### Genome sequencing data retrieval from plant pathogenic nematodes

The publically available genomes of completely sequenced plant pathogenic nematodes, *G. pallida* from Wellcome Trust Sanger Institute (ftp://ftp.sanger.ac.uk/pub/project/pathogens/Globodera/pallida/Gene_Predictions), *M. floridensis* from Nematode Genomes from the Blaxter lab, University of Edinburgh (http://nematodes.org/genomes/meloidogyne_floridensis/) and the remaining three i.e. *B. xylophilus* (http://www.wormbase.org/species/b_xylophilus#01--10), *M. hapla* (http://www.wormbase.org/species/m_hapla#01--10) and *M. incognita* (http://www.wormbase.org/species/m_incognita#01--10) from WormBase, were downloaded and further processed through the pipeline for the identification of genes encoding CWDEs.

### *In silico* identification of genes encoding cell wall degrading enzymes (CWDEs)

The amino acid sequence of conserved domains associated with the CWDEs were downloaded from the CAZy database (http://www.cazy.org/) [11] and used to create a HMM profile with HMMER v3.1b1 package (http://www.ebi.ac.uk/Tools/hmmer/). Each of the downloaded plant pathogenic nematode proteomes was searched for the presence of CWDEs using the hmmsearch program of HMMER package. Additionally, nematode proteomes were further screened with a Blast similarity search using the protein sequences downloaded from the CAZy database as query. The independently identified protein sequences from both analyses were pooled together, checked for the redundancy and the redundant protein sequences were removed from further analyses. The analysis of AA enzymes was done by downloading the representative protein sequences for each of the 13 AA enzyme sub-classes and performing the BlastP similarity search.

### Validation of mining pipeline

To validate the identification, all the non-redundant putative CWDE related protein sequences (GHs, PLs and AAs) were analyzed for the presence of conserved domains using NCBI’s conserved domain database (http://www.ncbi.nlm.nih.gov/Structure/bwrpsb/bwrpsb.cgi) [72] and Pfam database (http://pfam.xfam.org/) [73]. Any protein sequence without a conserved domain or with a conserved domain not related to CWDEs was eliminated from the further study.

### Common CWDE gene families across plant pathogenic nematodes

All the identified CWDE gene families from the five species of plant pathogenic nematodes were compared with each other to identify the gene families which are present in all the species. To visualize the comparison, a venn diagram was generated using the freely accessible online tool (http://bioinformatics.psb.ugent.be/webtools/Venn/).

### Transcript abundant analysis of CWDEs across the life cycle of *G. pallida*

Publically available transcriptome datasets (PRJEB2896) covering the entire life-cycle (invasive larval stage J2, adult male, 1, 7, 14, 28 and 35 days post infection (dpi)) of an important plant pathogenic nematode *G. pallida,* were downloaded from the NCBI’s short read archive (SRA) database (http://www.ncbi.nlm.nih.gov/Traces/study/?acc=ERP001236) (Additional file 2: Table S4). Sequence Read Archive (SRA) files of all the stages were mapped on *G. pallida*’s CWDE encoding genes using the QSeq program of DNASTAR Lasergene package (http://www.dnastar.com/t-nextgen-qseq.aspx). To visualize the transcript abundance, a hierarchical clustering heat map was generated using the self-normalized RPKM (reads per kilobase per million reads) values calculated by the QSeq program.

### Development of Nematode’s CWDE database

To ensure the availability of all the CWDEs identified from the plant pathogenic nematodes, a Nematode’s cell wall degrading enzyme database was created using the Microsoft’s expression web 4 which is available for public access at http://www.pssc.ttu.edu/ncwde/index.html. The sequence data are available for download in the fasta format.

## Additional files

Additional file 1: Table S1. List of identified CWDE encoding genes distributed into their respective gene families. (XLSX 14 kb) Additional file 2: Table S2. Details of Auxiliary Activity (AA) enzymes present in the plant pathogenic nematodes. Table S3 Species wise details of identified Carbohydrate Binding Modules (CBMs) in the various cell wall degrading enzymes. Table S4 Details of the downloaded SRA files source from different stages of the life-cycle of *G. pallida* for use in expression analysis. Table S5 Details of transcriptome data mapping on the different stages of the life-cycle of *G. pallida*. (DOCX 33 kb)

## Abbreviations

CWDEs: Cell wall degrading enzymes
HMM: Hidden markov models
SRA: Sequence read archive
CAZy: Carbohydrate active enZymes database
CAZymes: Carbohydrate active enZymes
GH: Glycoside hydrolases
PL: Polysaccharide lyases
GT: Glycosyl transferases
CE: Carbohydrate esterases
Pfam: Protein families
CDD: Conserved domain databases
RPKM: Reads per kilobase per million reads
NCWDE: Nematode cell wall degrading enzymes
AA: Auxiliary activity
