# Supplementary material for: Genome wide comprehensive analysis and web resource development on cell wall degrading enzymes from phyto-parasitic nematodes
Source: BMC Plant Biol. 2015 Aug 1;15:187. doi: 10.1186/s12870-015-0576-4 (PMC4521475; doi:10.1186/s12870-015-0576-4)
Supplement: Additional file 2: Table S2. — Details of Auxiliary Activity (AA) enzymes present in the plant pathogenic nematodes. Table S3 Species wise details of identified Carbohydrate Binding Modules (CBMs) in the various cell wall degrading enzymes. Table S4 Details of the downloaded SRA files source from different stages of the life-cycle of G. pallida for use in expression analysis. Table S5 Details of transcriptome data mapping on the different stages of the life-cycle of G. pallida. (DOCX 33 kb) [file 12870_2015_576_MOESM2_ESM.docx]

**Table S2** Details of Auxiliary Activity (AA) enzymes present in the plant pathogenic nematodes.

| **AA Family** | **Enzymatic activity** | **Species** | **Genes** |
| --- | --- | --- | --- |
| AA1 | Multi-copper oxidase | *B. xylophilus* | BUX.s01281.17;  BUX.s00116.660;  BUX.s00116.661 |
|  |  | *G. pallida* | GPLIN_001134500; GPLIN_001134600;  GPLIN_000667800 |
|  |  | *M. hapla* | MhA1_Contig1431.frz3.fgene3 |
| AA3 | GMC oxido-reductase | *B. xylophilus* | BUX.s01097.6 |
|  |  | *G. pallida* | GPLIN_000059200 |
|  |  | *M. floridensis* | mf.nMf.1.1.1.t10871-RA; mf.nMf.1.1.1.t22059-RA |
|  |  | *M. hapla* | MhA1_Contig32.frz3.gene8 |
|  |  | *M. incognita* | Minc04005  Minc06200 |
| AA4 | Vanillyl alcohol oxidase | *B. xylophilus* | BUX.s01259.99 |
| AA7 | Gluco-oligosaccharide oxidase | *G. pallida* | GPLIN_000227700; GPLIN_000540100;  GPLIN_000562000 |

**Table S3** Species wise details of identified Carbohydrate Binding Modules (CBMs) in the various cell wall degrading enzymes. (Abbreviations- GH2: β-Galactosidase; GH5; Endo-β-1,4-glucanase; GH18: Chitinase).

| **Species** | **Gene** | **CWDE Family** | **CBM identified** |
| --- | --- | --- | --- |
| *B. xylophilus* | BUX.s00422.469 | GH18 | CBM14 |
| *G. pallida* | GPLIN_000536400 | GH5 | CBM2 |
|  | GPLIN_000616300 | GH5 | CBM2 |
|  | GPLIN_000694900 | GH5 | CBM2 |
|  | GPLIN_000779200 | GH5 | CBM2 |
|  | GPLIN_000891100 | GH18 | CBM14 |
| *M. floridensis* | mf.nMf.1.1.1.t06892-RA | GH18 | CBM14 |
| *M. hapla* | MhA1_Contig1278.frz3.fgene1 | GH5 | CBM2 |
|  | MhA1_Contig344.frz3.gene3 | GH18 | CBM14 |
| *M. incognita* | Minc09446a | GH5 | CBM2 |
|  | Minc09446b | GH5 | CBM2 |
|  | Minc00169a | GH5 | CBM2 |
|  | Minc00169b | GH5 | CBM2 |
|  | Minc09298a | GH5 | CBM2 |
|  | Minc09298c | GH5 | CBM2 |
|  | Minc14047a | GH5 | CBM2 |
|  | Minc14047b | GH5 | CBM2 |
|  | Minc03287 | GH5 | CBM2 |
|  | Minc14048 | GH5 | CBM2 |
|  | Minc00168 | GH5 | CBM2 |
|  | Minc03286 | GH5 | CBM2 |
|  | Minc13221a | GH5 | CBM2 |

**Table S4** Details of downloaded SRA files source from the different stages of life-cycle of *G. pallida* for use in expression analysis.

| **Run name** | **Sample name** | **Stage** |
| --- | --- | --- |
| ERR202422 | SAMEA1570099 | Globodera_pallida_adult_males |
| ERR202423 | SAMEA1570103 | Globodera_pallida_14dpi_young_females |
| ERR202424 | SAMEA1570091 | Globodera_pallida_J2 |
| ERR202425 | SAMEA1570086 | Globodera_pallida_7dpi |
| ERR202426 | SAMEA1570105 | Globodera_pallida_1dpi |
| ERR202427 | SAMEA1570110 | Globodera_pallida_28dpi |
| ERR202428 | SAMEA1570114 | Globodera_pallida_35dpi |

| **Table S5** Details of transcriptome data mapping on the different stages of life-cycle of *G. pallida*. | | | | | | | |
| --- | --- | --- | --- | --- | --- | --- | --- |
| **Name** | **14_dpi** | **21_dpi** | **28_dpi** | **35_dpi** | **7_dpi** | **Adult_male** | **J2** |
| GPLIN_000300000 | 10.579 | 13.026 | 13.186 | 13.123 | 13.792 | 11.135 | 10.262 |
| GPLIN_001345700 | 3.798 | 0.953 | 0.953 | 0.953 | 0.953 | 5.713 | 4.996 |
| GPLIN_000523800 | 12.959 | 11.719 | 12.938 | 12.646 | 12.909 | 15.198 | 10.621 |
| GPLIN_000536400 | 8.373 | 9.820 | 8.711 | 8.899 | 9.041 | 17.170 | 18.446 |
| GPLIN_000545300 | 17.556 | 13.938 | 12.343 | 12.447 | 14.954 | 15.291 | 8.510 |
| GPLIN_000552400 | 7.677 | 9.585 | 11.063 | 10.918 | 8.640 | 9.300 | 13.189 |
| GPLIN_001416500 | 8.520 | 9.061 | 9.370 | 9.302 | 8.996 | 6.143 | 6.600 |
| GPLIN_000616300 | 8.323 | 9.979 | 8.559 | 9.643 | 7.155 | 14.256 | 8.215 |
| GPLIN_000635000 | 13.200 | 14.309 | 14.192 | 14.104 | 12.312 | 9.036 | 5.496 |
| GPLIN_000694900 | 0.953 | 0.953 | 0.953 | 0.953 | 0.953 | 6.315 | 3.200 |
| GPLIN_000734800 | 0.953 | 0.953 | 0.953 | 0.953 | 0.953 | 0.953 | 0.953 |
| GPLIN_000142900 | 8.680 | 4.486 | 8.998 | 9.045 | 9.457 | 11.858 | 11.350 |
| GPLIN_000143000 | 8.247 | 6.068 | 4.557 | 7.638 | 9.616 | 12.260 | 13.411 |
| GPLIN_000755100 | 3.610 | 0.953 | 0.953 | 0.953 | 2.249 | 15.735 | 4.088 |
| GPLIN_000755200 | 5.109 | 5.737 | 0.953 | 4.541 | 2.235 | 16.630 | 4.582 |
| GPLIN_000779000 | 2.082 | 1.192 | 0.953 | 0.953 | 4.078 | 4.235 | 5.482 |
| GPLIN_000779200 | 3.455 | 3.112 | 2.338 | 2.916 | 5.766 | 6.190 | 10.230 |
| GPLIN_000827200 | 4.012 | 0.953 | 0.953 | 5.248 | 4.535 | 5.964 | 5.747 |
| GPLIN_001508200 | 15.934 | 15.933 | 15.612 | 15.767 | 14.296 | 13.604 | 10.105 |
| GPLIN_000933200 | 7.388 | 8.434 | 7.486 | 7.238 | 7.643 | 16.350 | 18.369 |
| GPLIN_000943500 | 11.782 | 12.813 | 12.826 | 12.966 | 12.205 | 13.090 | 10.931 |
| GPLIN_000943900 | 11.878 | 12.740 | 12.500 | 12.211 | 12.798 | 12.118 | 10.677 |
| GPLIN_000031200 | 0.953 | 0.953 | 0.953 | 0.953 | 2.602 | 8.272 | 0.953 |
| GPLIN_001007400 | 14.544 | 15.517 | 15.784 | 15.844 | 14.971 | 12.771 | 9.615 |
| GPLIN_001111200 | 2.057 | 7.344 | 3.040 | 4.448 | 4.758 | 10.920 | 15.725 |
| GPLIN_001111300 | 0.953 | 0.953 | 4.019 | 0.953 | 4.012 | 10.060 | 14.620 |
| GPLIN_001185800 | 6.674 | 6.427 | 6.470 | 7.352 | 7.932 | 7.631 | 8.358 |
| GPLIN_000304900 | 7.402 | 4.820 | 3.462 | 6.483 | 7.821 | 6.210 | 7.123 |
| GPLIN_000309300 | 12.185 | 11.419 | 11.699 | 11.729 | 12.785 | 12.666 | 9.625 |
| GPLIN_000309600 | 12.019 | 11.560 | 12.437 | 12.333 | 12.855 | 13.723 | 11.412 |
| GPLIN_000313600 | 4.231 | 0.953 | 7.160 | 4.453 | 2.410 | 11.130 | 14.085 |
| GPLIN_001215600 | 3.238 | 0.953 | 4.636 | 0.953 | 6.505 | 12.319 | 14.579 |
| GPLIN_000361800 | 12.287 | 11.779 | 11.705 | 11.194 | 13.625 | 11.867 | 8.492 |
| GPLIN_000361900 | 13.582 | 13.648 | 13.752 | 13.464 | 14.276 | 12.847 | 15.203 |
| GPLIN_000370900 | 8.577 | 5.883 | 7.329 | 4.811 | 8.734 | 0.953 | 4.069 |
| GPLIN_000412400 | 15.002 | 15.190 | 14.856 | 14.837 | 16.493 | 14.037 | 10.544 |
| GPLIN_000412500 | 14.042 | 14.483 | 15.414 | 15.545 | 14.776 | 13.411 | 10.049 |
| GPLIN_000412600 | 12.567 | 13.674 | 13.994 | 14.010 | 13.327 | 11.729 | 8.229 |
| GPLIN_001010600 | 0.953 | 0.953 | 0.953 | 0.953 | 0.953 | 0.953 | 0.953 |
| GPLIN_000538800 | 4.946 | 0.953 | 6.666 | 6.929 | 5.879 | 9.819 | 6.765 |
| GPLIN_000542000 | 8.897 | 12.914 | 14.060 | 13.658 | 8.361 | 8.158 | 5.854 |
| GPLIN_000562100 | 7.847 | 9.828 | 14.260 | 14.379 | 10.243 | 11.176 | 7.828 |
| GPLIN_000654900 | 10.607 | 11.400 | 11.601 | 11.849 | 11.914 | 11.426 | 10.497 |
| GPLIN_000699900 | 10.341 | 11.066 | 11.655 | 11.583 | 11.056 | 12.429 | 10.908 |
| GPLIN_000707700 | 8.618 | 9.859 | 10.701 | 10.726 | 11.136 | 10.220 | 8.762 |
| GPLIN_000183600 | 9.146 | 8.426 | 10.493 | 10.759 | 9.812 | 10.446 | 9.626 |
| GPLIN_000891100 | 10.050 | 11.425 | 12.637 | 12.085 | 10.800 | 10.242 | 8.217 |
| GPLIN_001532300 | 2.644 | 0.953 | 0.953 | 5.597 | 4.969 | 7.937 | 7.750 |
| GPLIN_000201500 | 12.583 | 12.906 | 12.945 | 12.855 | 13.333 | 13.029 | 10.287 |
| GPLIN_001541300 | 9.579 | 10.906 | 11.907 | 11.298 | 11.631 | 9.875 | 8.671 |
| GPLIN_001566500 | 5.793 | 6.553 | 7.866 | 7.201 | 7.313 | 5.782 | 5.507 |
| GPLIN_001120500 | 11.416 | 12.082 | 11.599 | 11.721 | 11.606 | 11.579 | 7.853 |
| GPLIN_001120600 | 9.106 | 9.237 | 8.176 | 9.920 | 10.877 | 10.172 | 6.284 |
| GPLIN_001591000 | 8.523 | 9.707 | 7.583 | 9.744 | 10.319 | 9.124 | 3.326 |
| GPLIN_001215200 | 0.953 | 0.953 | 0.953 | 0.953 | 0.953 | 0.953 | 0.953 |
| GPLIN_001228900 | 0.953 | 0.953 | 3.974 | 0.953 | 0.953 | 6.556 | 5.201 |
| GPLIN_000302800 | 12.631 | 12.661 | 12.353 | 12.480 | 12.750 | 14.085 | 11.674 |
| GPLIN_000907600 | 13.300 | 14.146 | 14.091 | 14.197 | 14.104 | 12.739 | 11.982 |
| GPLIN_000960200 | 12.039 | 12.140 | 11.735 | 11.647 | 12.758 | 13.237 | 11.134 |
| GPLIN_000906800 | 15.090 | 15.025 | 13.964 | 14.245 | 15.897 | 8.904 | 7.147 |
| GPLIN_001033700 | 14.836 | 15.116 | 14.517 | 14.854 | 14.875 | 12.980 | 11.894 |
| GPLIN_001068600 | 14.310 | 15.181 | 13.901 | 13.521 | 15.114 | 11.316 | 8.428 |
| GPLIN_001238800 | 10.519 | 11.714 | 12.865 | 12.670 | 11.948 | 11.917 | 9.219 |
| GPLIN_001068700 | 12.426 | 13.502 | 12.012 | 11.998 | 13.195 | 8.136 | 6.633 |
| GPLIN_000618600 | 9.982 | 11.029 | 12.000 | 11.749 | 10.420 | 9.074 | 6.400 |
| GPLIN_000757800 | 7.635 | 6.923 | 6.928 | 6.600 | 7.175 | 6.985 | 4.309 |
| GPLIN_000024800 | 13.325 | 13.736 | 12.659 | 12.591 | 13.878 | 12.820 | 12.641 |
| GPLIN_000949800 | 9.764 | 10.421 | 10.042 | 9.478 | 11.833 | 7.380 | 6.189 |
| GPLIN_000950400 | 9.415 | 9.828 | 9.921 | 9.097 | 11.993 | 5.876 | 6.044 |
| GPLIN_001068900 | 10.865 | 10.002 | 8.843 | 8.680 | 12.794 | 6.623 | 3.635 |
| GPLIN_001106700 | 9.443 | 9.038 | 8.951 | 9.636 | 9.247 | 9.095 | 9.243 |
| GPLIN_001106800 | 9.920 | 9.472 | 8.900 | 10.655 | 8.970 | 8.728 | 9.318 |
| GPLIN_000026600 | 10.322 | 11.636 | 12.269 | 12.412 | 10.011 | 11.524 | 8.950 |
| GPLIN_000273700 | 13.413 | 15.231 | 14.944 | 14.895 | 14.429 | 13.098 | 11.480 |
| GPLIN_000690600 | 10.891 | 12.166 | 12.568 | 12.690 | 12.157 | 11.129 | 9.304 |
| GPLIN_001115300 | 12.338 | 13.122 | 13.152 | 13.358 | 13.397 | 12.670 | 11.255 |
| GPLIN_000377400 | 9.109 | 10.159 | 9.578 | 9.901 | 11.276 | 12.085 | 11.303 |
| GPLIN_001110600 | 11.956 | 12.379 | 12.765 | 12.706 | 12.249 | 10.632 | 8.651 |
| GPLIN_001383300 | 11.566 | 11.784 | 12.000 | 12.108 | 11.406 | 9.662 | 9.018 |
| GPLIN_000238800 | 9.945 | 10.565 | 10.563 | 11.046 | 10.675 | 10.269 | 8.568 |
| GPLIN_001115200 | 11.153 | 11.008 | 11.171 | 10.736 | 10.926 | 12.017 | 9.188 |
| GPLIN_000674600 | 8.576 | 8.288 | 9.322 | 8.995 | 9.208 | 4.079 | 6.464 |
| GPLIN_000594300 | 11.539 | 12.538 | 12.489 | 12.457 | 12.784 | 11.860 | 11.336 |
| GPLIN_000689200 | 12.242 | 12.371 | 12.361 | 12.434 | 12.392 | 12.074 | 9.939 |
| GPLIN_001517700 | 11.410 | 12.048 | 12.335 | 12.081 | 12.067 | 10.959 | 8.414 |
| GPLIN_001123100 | 11.878 | 12.317 | 12.277 | 12.457 | 12.404 | 11.383 | 10.133 |
| GPLIN_001404500 | 9.336 | 7.514 | 7.833 | 8.578 | 10.601 | 10.556 | 5.717 |
| GPLIN_001423900 | 5.259 | 0.953 | 0.953 | 3.775 | 5.317 | 6.549 | 4.637 |
| GPLIN_000902500 | 4.262 | 4.149 | 0.953 | 1.953 | 2.910 | 4.897 | 9.013 |
| GPLIN_000194200 | 7.664 | 9.171 | 11.188 | 11.543 | 8.995 | 9.592 | 6.021 |
| GPLIN_001248000 | 6.139 | 6.803 | 5.826 | 6.568 | 5.497 | 0.953 | 15.777 |
| GPLIN_000227600 | 13.223 | 13.071 | 13.569 | 13.376 | 13.422 | 14.373 | 11.841 |
| GPLIN_000467400 | 4.574 | 0.953 | 0.953 | 4.795 | 5.922 | 11.255 | 15.353 |
| GPLIN_000673000 | 6.496 | 7.276 | 0.953 | 6.167 | 7.792 | 0.953 | 6.662 |
| GPLIN_000142600 | 0.953 | 0.953 | 0.953 | 0.953 | 0.953 | 8.939 | 14.088 |
| GPLIN_000294400 | 4.383 | 0.953 | 0.953 | 0.953 | 4.390 | 0.953 | 8.234 |
| GPLIN_000294500 | 4.517 | 3.689 | 6.559 | 5.493 | 6.189 | 4.682 | 9.226 |
| GPLIN_000322300 | 6.058 | 4.707 | 5.518 | 4.926 | 6.883 | 5.507 | 4.150 |
| GPLIN_000412300 | 7.592 | 11.478 | 13.166 | 12.804 | 8.333 | 10.757 | 13.867 |
| GPLIN_001452200 | 0.953 | 0.953 | 0.953 | 0.953 | 0.953 | 0.953 | 0.953 |
